# Increased complexity of social category markers leads to diverse rule-based categorizations and reduced intergroup bias

**DOI:** 10.1098/rspb.2025.0659

**Published:** 2025-07-30

**Authors:** Uri Hertz

**Affiliations:** ^1^ Department of Cognitive Sciences, University of Haifa, Haifa, Israel; ^2^ Institute of Information Processing and Decision Making, University of Haifa, Haifa, Israel

**Keywords:** social categories, social cognition, intergroup bias, categorization, social markers

## Abstract

Social categories are key to human social life, often leading to intergroup bias and stereotypes. While traditional studies use binary social markers, real-world markers are more complex. This study explored whether such complexity results in complex social categories or less cognitively demanding binary rules. Participants (*n* = 784, prolific) played a multiplayer video game where they competed with other players for stars and could remove other players from their path by zapping them, with manipulated player appearances: binary colour, multidimensional shape and colour or a continuous colour gradient. In all conditions, participants showed an intergroup bias, zapping players who were similar to them less than dissimilar players. This effect was reduced when social markers were complex. However, the group pattern was shown to be driven by participants’ idiosyncratic binary rules in their behaviour, revealed using clustering analysis, with rule diversity increasing with social marker complexity. Notably, some participants showed differential treatment of the most dissimilar players, while others singled out intermediate-colour players, resulting in less salient social categories overall. These findings provide a mechanistic understanding of social category formation and the way exposure to complex markers of social identity scales up to shape collective intergroup bias.

## 1. Introduction

Social categories are a fundamental aspect of human social life. They determine cooperation and competition patterns [1–3], how we learn about others’ beliefs and behaviour [4] and our own self-identity [5,6]. A well-studied distinction is between people belonging to one’s group (the ingroup) and people who do not belong to it (the out-group), leading to a binary view of the social world as two opposed categories, and to polarization [1,2,5]. Distinct social categories were also shown to be the basis of discriminative behaviour, based on social dimensions such as socioeconomical status, gender and ethnic background [7,8]. While social categories may rely on salient markers of social groups, it is often the case that social markers are more complex [9–12]. Social markers can vary on multiple dimensions, such as political ideology and religious belief [13], and lie on a continuum between different binary categories [14]. However, it is not clear how such complex social markers shape the emergence of social categories. This work takes a cognitive approach to the problem of categorization [15,16] and examines how cognitive processes shape social categorization and intergroup bias in environments with complex social markers.

Studies of social categorization are often designed to examine processes where social categories are salient, like intergroup bias, polarization and stereotypes [4,17–19]. For example, to study how people treat and react to outgroup members, clear group identification such as political ideology, race, gender or some arbitrary markers are primed and introduced before evaluating participants’ responses. This can allow detection of intergroup favouritism [2], coalitional thinking [20], perception of outgroups [21] and social learning biases [4]. When studying perception of stereotype and stereotype change, well-defined social categories are usually presented. This can help establish the content of such stereotype [22], stereotype change [23] and the way people use stereotypes to predict others’ traits and interact with them [24]. In such cases, it is important to establish early on in a clear manner the social categories with which the protagonists of the experimental vignettes are affiliated.

However, social categories are not always so well defined and may rely on multiple social markers [9,11,13]. People may belong to multiple groups which may intersect and challenge clear-cut categorization. Studies where group membership is defined by more than one category (age and gender, for example) show that people use multiple categories when judging similarity to others [25] and are more generous in money splitting decisions (i.e. the dictator game) towards more similar players than towards dissimilar players [26]. Other works show that when judging traits of individuals belonging to multiple social categories, people do not use the additive rule, but rather draw from the negative and extreme stereotypic traits more than from positive traits [27]. Finally, context and attention can change how people use social markers to form context-relevant social categories, focussing on either one category or the other [10,21]. These findings suggest that people can indeed take into account multiple dimensions when performing social categorizations, but that this process may be demanding, leading them to focus on one dimension whenever possible. This is similar to findings in non-social categorization, where participants are shown to be able to rely on multiple dimensions for categorization but make mistakes indicating over-reliance on a single dimension [16,28]. This suggests that perhaps a cognitive limitation may contribute to the social categorization problem.

Social categories may also lie on a continuum, where the boundaries between two distinct categories are not clear or where intermediate categories lie between two opposing categories [29]. For example, gender includes non-binary categories that lie between male and female, and race includes people with mixed origins [30]. When asked to categorize social stimuli that lie on continuum scales, participants usually tend to use binary categorization, for example categorizing faces as either very male/female or very black/white, rather than using intermediate categories [14,29,31]. When reaching judgements about people belonging to intermediate groups, people tend to view these groups as not real, and negatively evaluate them [30]. In the political arena, people were shown to prefer individuals with extreme views and negatively evaluate those with moderate or ambivalent opinions [32,33]. These findings suggest that while social markers may lie on a continuum, people tend to either use binary categorization rules or single out intermediate groups. In non-social perceptual categorization, people can show continuous categorization, for example based on similarity [15], but tend to follow a rule-based categorization process when possible [34], as keeping track of continuous similarity distance is more demanding than heuristic-based categorization.

The studies discussed above show that highlighting different social markers shapes the social categories people use, and how these affect predictions of others’ traits. However, it is not clear how these shape social interactions and intergroup bias. A well-known contact theory approach [35] suggests that direct interaction between members of different groups can lead to a reduction in intergroup bias, as one’s experience can override prejudice based on stereotypes. However, these findings seem to be limited to interactions in the context of common goal and cooperation [36]. Recent work shows that intergroup bias can be maintained even when ingroup and outgroup members behave in a similar way, due to asymmetric learning mechanisms, which amplify negative behaviour of outgroups [4,37]. However, these works relied on distinct social markers of group identity, just like other works on polarization, and it is not clear how complex social markers shape the emergence of intergroup bias through social categorization. One prediction is that intergroup bias will emerge even in complex settings, as people will use binary-rule-based categorization. Another is that a continuous, similarity-based, rule will be used. However, it may also be that when social markers are complex, experience during interpersonal interactions will determine behaviour, as predicted by contact theory.

**Figure 1.**
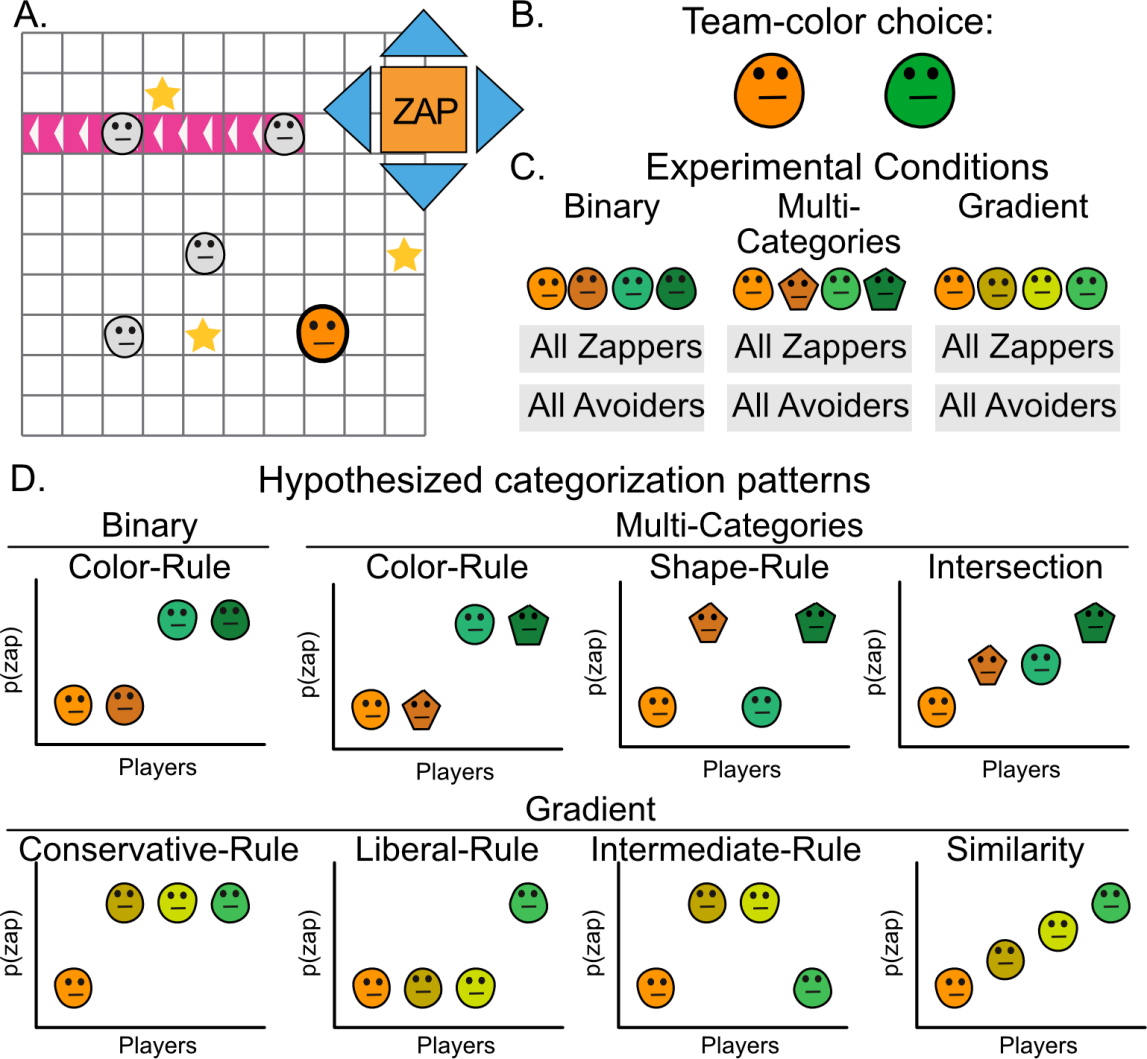
Experimental design and hypothesized social categorization patterns. (A) Participants played a multiplayer star-harvest game online, where they could move across a grid and collect stars. They could also shoot a ray that zaps other players and sends them to a time out zone (pink ray). (B) Before beginning the game, participants were asked to choose a team colour for their avatar. (C) Participants played the game with four other players. These players’ actions were governed by one of two algorithms—avoider or zapper. Avoider players did not zap other players, while zapper players zapped players who were on their path to a star. Participants were tested in three experimental conditions: Binary, where players’ colours belonged to either the green or orange group; multi-categories, where players differed in colour and shape; and gradient, where players’ colours lay on a continuous gradient. (D) Hypothesized patterns of zapping behaviour in the three experimental conditions.

The current work aims to evaluate how people form social categories when faced with complex social markers. Specifically, it asks whether people employ binary rules, categorizing others to either ingroup or outgroup, or whether they are able to accommodate more subtle continuous distinctions. To examine the social categorization problem, participants played a multiplayer video-game, where they could move about a grid, collect stars and shoot a ray that zaps other players and sends them to a time out zone (figure 1) [4,38]. Each game started with a minimal-group manipulation, where participants choose their avatar’s colour from one of two groups. In three experiments, the appearances of other players’ avatars were manipulated to examine how social markers complexity affect participants behaviour. In the first experiment, avatars had distinct colours that belonged to one of the two groups, thus making it easy to form a binary social categorization [4]. In the multi-categories experiment, participants played with players who had distinct group colours but an additional difference in the shape dimension that was not used in the minimal-group manipulation. In the gradient experiment, players’ colour lay on a continuous scale between the two groups’ colours, with two players showing intermediate shades.

This experimental design allowed detection of the categorization patterns underlying participants’ zapping behaviour, and how changes in social markers’ complexity shape these patterns. One such pattern is a binary rule, where the other players belong to one group or another, while another pattern is similarity-based categorization, where treatment of other players changes gradually (figure 1). In addition, it is possible to detect a variety of binary rules, as one may parse the world in different ways. For example, one may employ a liberal rule, where very dissimilar others are treated differently than more similar players, or a conservative rule where very similar others are treated differently than other players. It can also allow detection of more complex categorization patterns, such as negative treatment of intermediate categories in the gradient experiment. Finally, this design allows multiple rules to emerge and can therefore be used to evaluate whether categorization is idiosyncratic or similar across participants and whether it is apparent on the group level.

## 2. Methods

### (a) Participants

Participants were recruited through the prolific online platform and performed the experiment online. Previous work [4] indicated an expected effect size of partial η^2^ 0.07. To reach 90% power with *α* of 0.05, sample size of 105 participants per experimental behaviour condition (all-zappers/all-avoiders) was needed. I therefore set out to recruit 250 participants for the multi-categories and another 250 participants for the gradient conditions, to account for drop-off rates. As some participants did not complete the task, I analysed data from 246 participants in the multi-categories condition and 241 participants in the gradient condition. The multi-categories included 118 female participants (mean age ± s.d. = 35 ± 10), 123 male participants (age = 34.2 ± 10.6), 2 non-binary participants (aged 31 and 52) and 3 participants who did not disclose their gender (aged 32,34,43). The gradient condition included 112 female participants (33.3 ± 10), 121 male participants (age 34 ± 10), 7 non-binary participants (aged 37.3 ± 14.2) and one participant who did not disclose their gender (aged 32). Participants received compensation of GBP 2.5 for their participation, and the experiment lasted around 15 min.

For the binary condition I used data collected in previous work [4]. It includes data from 297 participants in two experimental condition (all-zappers and all avoiders), comprising 149 female participants (mean age ± s.d. = 38 ± 12), 146 male participants (age = 39 ± 13.6) and 2 participants who did not disclose their gender (aged 26, 31). All details about recruitment, pre-registration and ethics approval are provided in the published article [4].

### (b) Experimental task

Participants played a version of the star harvest game [4,38] (figure 1). In this game, multiple players move about a two-dimensional grid one at a time and collect stars that appear from time to time. They can also choose to zap (shoot) a player instead of moving. When they zap, they send a ray in one of four directions. Whoever is caught in this ray is sent to a time out zone outside the grid for three turns. The task comprised 100 turns; each turn included a behaviour from all players, one after another.

Participants played this game with four other bot-players whose behaviour was governed by one of two algorithms—avoider or zapper. The avoider-algorithm players moved to collect stars when they were closest to a star and moved away from stars when another player was in their path, and never zapped. The zapper-algorithm players moved to collect stars when they were closest to a star and zapped players who were on their path to a star (for more details see the supplementary material of [38]). In each condition (binary/multi-categories/gradient), participants played in one of two behaviour conditions. In the all-zappers condition, all four bot-players were governed by a zapper algorithm. In the all-avoiders condition, all four bot-players were governed by an avoider algorithm.

Participants chose their avatar’s team colour before beginning the game, in line with previous work and minimal group manipulations [4,39] (figure 1). They had to choose one of six avatars, all with a round shape. Half were different shades of green, part of the green team and the other half different shades of orange, part of the orange team. Participants then continued to play the game itself. In the binary condition [4], participants played the game with two players represented by round green avatars, and two players represented by round orange avatars. In the multi-categories condition, participants played the game with a round green player, a pentagon green player, a round orange player and a pentagon orange player. In the gradient condition, participants played the game with players represented by round avatars, one of which was green and another orange, and the other two had shades between green and orange, determined using their colour values to lie along a linear distance between the green and orange.

Upon beginning the task participants were told that they were about to play a multiplayer game with four other players and were not given any further information about the identity of the other players. The participants were debriefed at the end of the game and were told that the other players were algorithmic players. Participants were provided with contact information of the researchers and were encouraged to contact them if they had any questions or concerns.

After completing the game, participants performed the star-allocation task. They were given ten extra stars, saw the avatars of all players and were asked to divide the stars between the four players.

### (c) Analyses

To evaluate participants’ social categorization, the analyses focussed on two measures—how participants allocated stars to the four players after the game, and their likelihood of zapping players during the game.

Three different analyses were used to examine likelihood to zap during the game. I pre-registered a mixed-effect logistic regression, with the participants’ decision to zap or not (1/0) in every trial in which they shared a column or row with another player, the target player, as the dependent variable. The independent variables included the similarity of the target player to the participant, which was defined differently in each social-markers condition. In the binary condition, avatar colour (same/different) was used. In the multi-categories condition, avatar colour (same/different), avatar shape (same/different) and their interaction as were used as dependent variables. In the gradient condition, a linear distance (0−3) was used to capture the gradual difference in avatar colours from similar to different. A quadratic distance (0−9) was also included, to account for nonlinear effects. Other independent variables were the current distance of the participant from the target player, the target player’s playing order in the game, and whether the participant was currently the closest to a star. All these variables were predicted to affect the participant’s decision whether to zap the target player or not. Participants’ individual baseline zapping rate was included as a random variable.

To explore how participants’ zap likelihood changed during the game, the experimental block was divided into four time-bins, each including 25 turns. For each player, the portion of times in which they zapped each of the other four players when they could (shared a column or row with them) during each time-bin was measured and used as a dependent variable in a a mixed-effect linear regression. Time-bins (0−3), player’s similarity (as described above, but without the quadratic variable in the gradient condition) and their interaction were used as independent variables. This analysis included both behaviour conditions, and therefore, we included a behaviour-condition (all-avoiders/all-zappers) and its interaction with time-bins as independent variables. Finally, participants’ individual baseline zapping rates and leaning slopes were included as random variables. This analysis was carried out separately for each social-markers condition.

A planned clustering analysis was carried out for each social-markers condition. Each participants’ likelihood to zap each of the four players was measured, ordered by their similarity to the participant, across the entire game and centred it by removing each player’s average zapping rate. All participants’ likelihood to zap were then scaled to have variance of 1 and average of 0, and these were used in a *k*-means analysis. Each participant comprised a single four-dimensional vector (likelihood to zap each of the four players). Silhouette analysis was used to evaluate the benefit of increased number of clusters, with the purpose of determining the optimal number of clusters to describe differences in each of the social-markers conditions [40], and it was possible to examine under which number of clusters this benefit peaked or reached a plateau. Finally, unscaled zapping behaviour of participants in the different clusters was examined to determine the differences between conditions.

For star allocation analysis, a linear regression analysis was pre-registered, with the number of stars as the dependent variable and the similarity of the players’ avatar to the participants’ avatar as the independent variable, as detailed above. This analysis was carried out independently for each behaviour-condition (all-zappers/all-avoiders) and each social-markers condition (binary/multi-categories/gradient).

Analyses were carried out using R statistical programming software version 4.3.1. Full information regarding the R packages used in the analyses is provided in the electronic supplementary material.

## 3. Results

### (a) Intergroup bias in zapping behaviour

The main behavioural measure in this study was participants’ zapping behaviour, measured as the percentage of times participants zapped another player when they had a chance (when they shared a column or row). Participants’ zapping behaviour towards all players was used as the dependent variable in a pre-registered mixed-effects logistic regression to evaluate their trial-by-trial decisions to zap a player, with the player’s similarity, the distance to the player, whether the participant is currently the closest to a star, and the player’s order in the task as independent variables. Similarity was modelled as colour (same/different) in the binary condition, colour (same/different), shape (same/different) and their interaction in the multi-categories condition and linear (0−3) and quadratic (0−9) similarity distance in the gradient condition. Additional experiment variables were included: the target player’s playing order, whether the participant was currently the closest player to a star, and the participant’s current distance from the target player.

**Figure 2.**
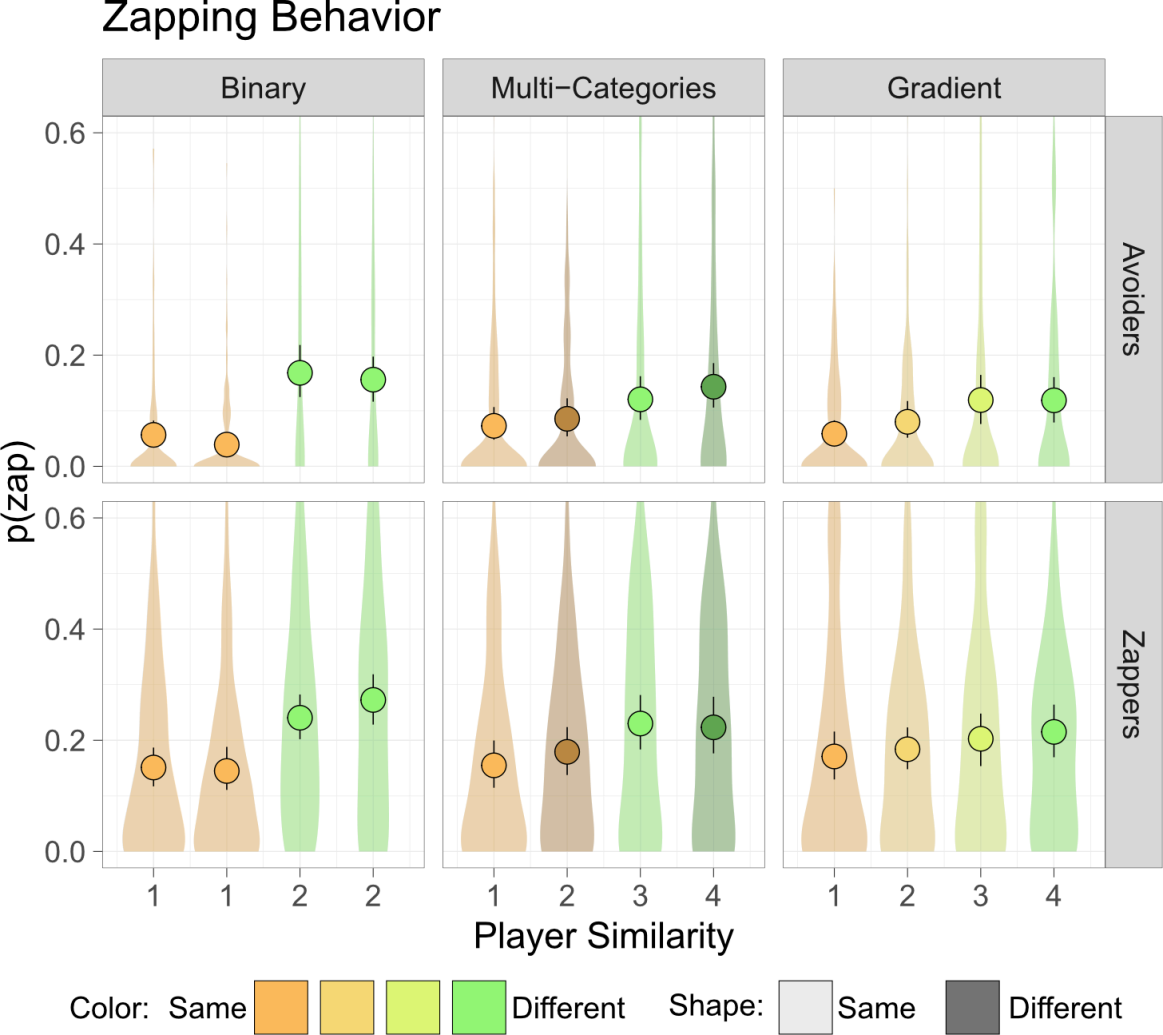
Intergroup bias in zapping behaviour. Participants’ likelihood to zap other players, *p*(zap), was dependent on the players’ similarity to the participant and on the players’ behaviour (zappers/avoiders). In the binary condition players were either the same/different colour than the player, in the multi-categories condition players also had similar/different shape as the participant, and in the gradient condition players’ colours lay on a scale. Circles represent the mean, lines represent bootstrap 99% confidence interval of the mean, and violins represent the distribution of individual responses.

In all experimental conditions, participants were significantly less likely to zap when they were the closest player to a star, indicating that they preferred gaining a star over zapping, and they were less likely to zap targets who were far away from them (figure 2; see full results for all conditions in the electronic supplementary materials). In the binary condition, participants were less likely to zap players who had the same avatar colour as them (all-avoiders: estimate ± s.e.: −1.44 ± 0.11, *z* = −13.42, *p* < 0.001, standard coefficient (SC): −1.44 [−1.66, −1.23], all-zappers: −0.75 ± 0.074, *z* = −10.10, *p* < 0.001, SC: −0.75 [-0.90, −0.60]). In the multi-categories condition, participants showed a similar colour effect (all-avoiders: −0.84 ± 0.15, *z* = −5.65, *p* < 0.001, SC: −0.84 [−1.14, −0.55], all-zappers: −0.39 ± 0.12, *z* = −3.39, *p* < 0.001, SC: −0.39 [−0.62, −0.17]). Participants were also marginally affected by players’ shape in the all-avoiders condition (−0.25 ± 0.13, *z* = −1.88, *p* = 0.06, SC: −0.25 [−0.52, 0.01]) but did not show a significant shape effect in the all-zappers condition, and did not show a significant interaction in both conditions. Finally, in the gradient condition, participants were sensitive to linear similarity-distance (all-avoiders: 0.43 ± 0.088, *z* = 4.936, *p* < 0.001, SC: 0.49 [0.29, 0.68], all-zappers: 0.15 ± 0.054, *z* = 2.83, *p* = 0.0047, SC: 0.17 [0.05, 0.29]). Participants also showed a significant negative quadratic-distance effect in the all-avoiders condition (−0.14 ± 0.063, *z* = −2.20, *p* = 0.027, SC: −0.21 [−0.39, −0.02]), indicating that they did not distinguish between players who were more than one similarity-distance from them.

As an exploratory evaluation of the difference in intergroup bias between the experimental conditions, I examined the differences in average zapping rates of the most similar and most dissimilar players (1 and 4 in figure 2) in each condition. Experimental condition (binary/multi-categories/gradient), the players’ similarity (one-fourth), and their interaction were included as independent variables, the players’ behaviour (all-zappers/all-avoiders) as another independent variable, and the participants’ baseline as random variables in a linear mixed-effects regression. Participants showed an overall intergroup bias effect (0.11 ± 0.012, *t*(780) = 9.27, *p* < 0.001, SC: 0.59 [0.46, 0.71]), in line with the previous analyses. There was also a significant interaction between conditions and player similarity, showing a reduction of intergroup bias effect in the multi-categories (−0.04 ± 0.017, *t*(780) = −2.30, *p* = 0.021, SC: −0.22 [-0.40, −0.03]) and gradient (−0.058 ± 0.018, t(780) = −3.30, *p* = 0.001, SC: −0.31 [−0.50, −0.13]) conditions. This shows that increased complexity of social markers led to reduction of intergroup bias.

### (b) Dynamic changes in intergroup bias

**Figure 3.**
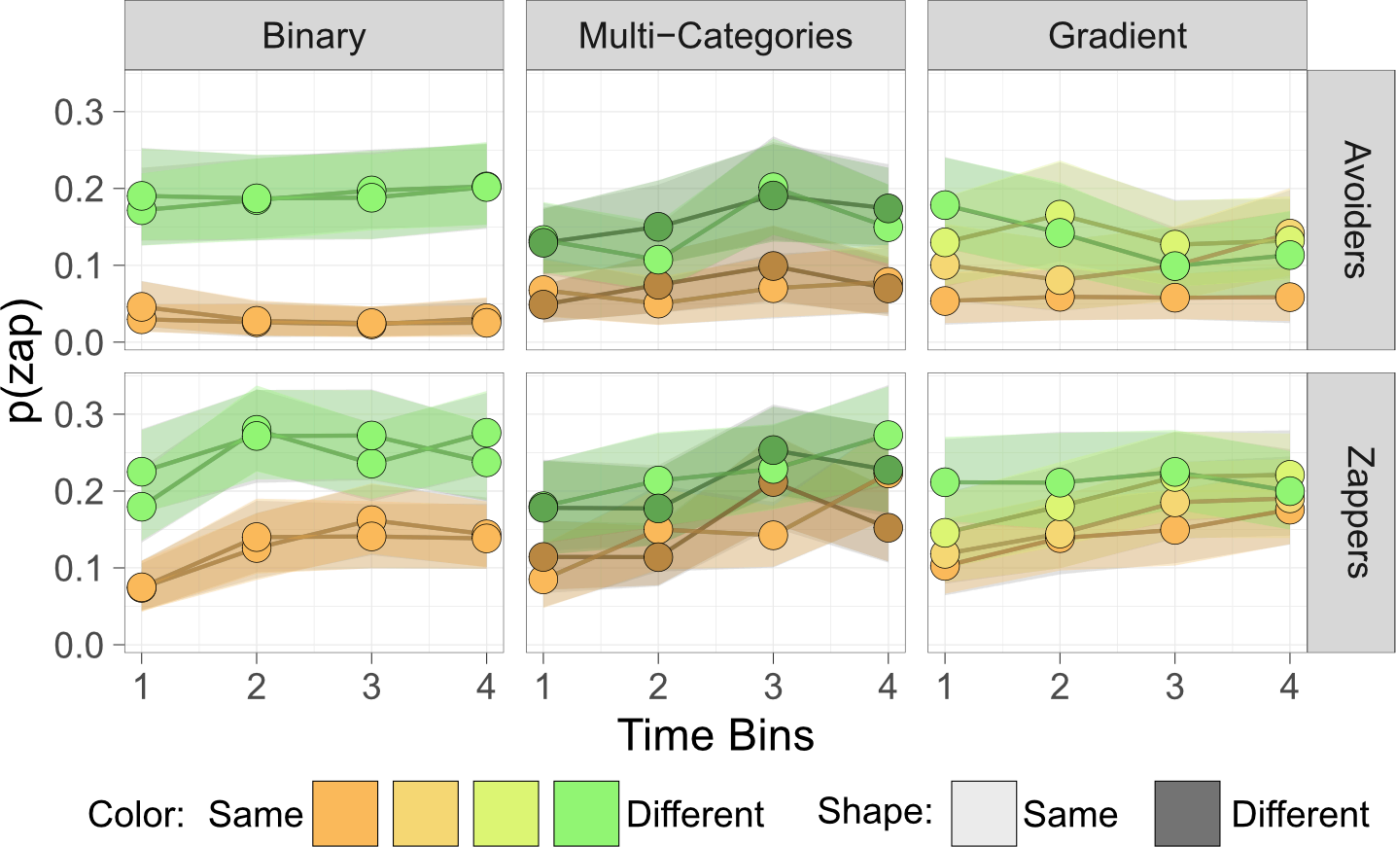
Dynamic changes in intergroup bias. Zapping behaviour was binned to four time points across the experimental blocks. Participants showed consistent intergroup bias in the binary and multi-categories conditions, but this bias diminished in the gradient condition. Dots represent the mean and shadows represent the bootstrap 99% confidence interval of the mean.

An exploratory, non-registered analysis examined the dynamics of intergroup bias throughout the experiment. In previous work, we showed that participants learned about other players’ zapping behaviour and adapted their behaviour accordingly, increasing their likelihood to zap players who were zappers, and that intergroup bias persisted even when all players displayed the same behaviour [4,38]. To examine learning patterns in the current experiment, the experimental block was divided into four time-bins and examined participants’ average zapping rate towards each of the other players (figure 3). Three mixed-effects linear regressions, one for each experimental condition (binary/multi-categories/gradient), were used with mean zap rates as the dependent variable, and similarity to player, time-bins (1–4), and their interactions as independent variables.

In the binary condition, there was a significant colour effect (−0.13 ± 0.018, *t*(3960) = −7.19, *p* < 0.001, SC: −0.50 [−0.55, −0.45]). There was no significant time-bins effect, but there was a significant interaction between time-bins and the all-zappers condition (0.018 ± 0.0067, *t*(3955) = 2.66, *p* = 0.0078, SC: 0.07 [0.02, 0.12]). These indicate that in the all-zappers condition participants increased their zapping behaviour over time, but not in the all-avoiders condition. There was no significant interaction between colour and time-bins, indicating persistence of intergroup bias over time (figure 3). In the multi-categories condition, there was a similar pattern of results, with a significant colour effect (−0.07 ± 0.02, *t*(3247) = −3.47, *p* < 0.001, SC: −0.28 [−0.34, −0.22]) but not a significant shape effect. The time-bins effect was not significant, but there was a significant interaction between time-bins and the all-zappers condition (0.019 ± 0.0073, *t*(3247) = −2.607, *p* = 0.009, SC: 0.08 [0.14, 0.02]). These indicate that in the all-zappers condition participants increased their zapping behaviour over time, but not in the all-avoiders condition. Finally, there was no significant interaction between colour or shape and time-bins, indicating persistence of intergroup bias over time.

A different pattern of zapping behaviour was found in the gradient condition. As before, there was a significant similarity effect (0.036 ± 0.006, *t*(3039) = 6.104, *p* < 0.001, SC: 0.10 [ 0.07, 0.13]), indicating an intergroup bias. The time-bins effect was not significant, but there was a significant interaction between the all-zappers condition and time-bins (0.02 ± 0.01, *t*(248) = 2.33, *p* = 0.020, SC: 0.08 [0.01, 0.15]), indicating that in the all-zappers condition participants increased their zapping behaviour over time, but not in the all-avoiders condition. Unlike the other conditions, in the gradient condition, I found a significant interaction between similarity and time-bins (−0.0081 ± 0.0032, *t*(3046) = −2.514, *p* = 0.012, SC: −0.04 [−0.07, −0.01]). This indicated that the similarity effect, which is an indicator of intergroup bias, decreased over time and that by the end of the experimental block participants did not differentiate between players based on similarity. While social markers of similarity and group affiliation initially played a role, participants overcame these priors and adapted their zapping behaviour to the players’ behaviour regardless of social markers.

### (c) Diverse rule-based categories in zapping behaviour

The aggregate results across participants could be the result of a single but noisy pattern, or the result of averaging many distinct patterns. For example, if a subgroup of participants in the multi-categories condition was sensitive only to avatars’ colours, while another subgroup was sensitive only to avatars’ shape, the resulting aggregated measure would show sensitivity to both colour and shape and the effect sizes would be determined by the relative size of each subgroup. To better understand participants’ zapping behaviour in our task, a planned clustering analysis was conducted.

**Figure 4.**
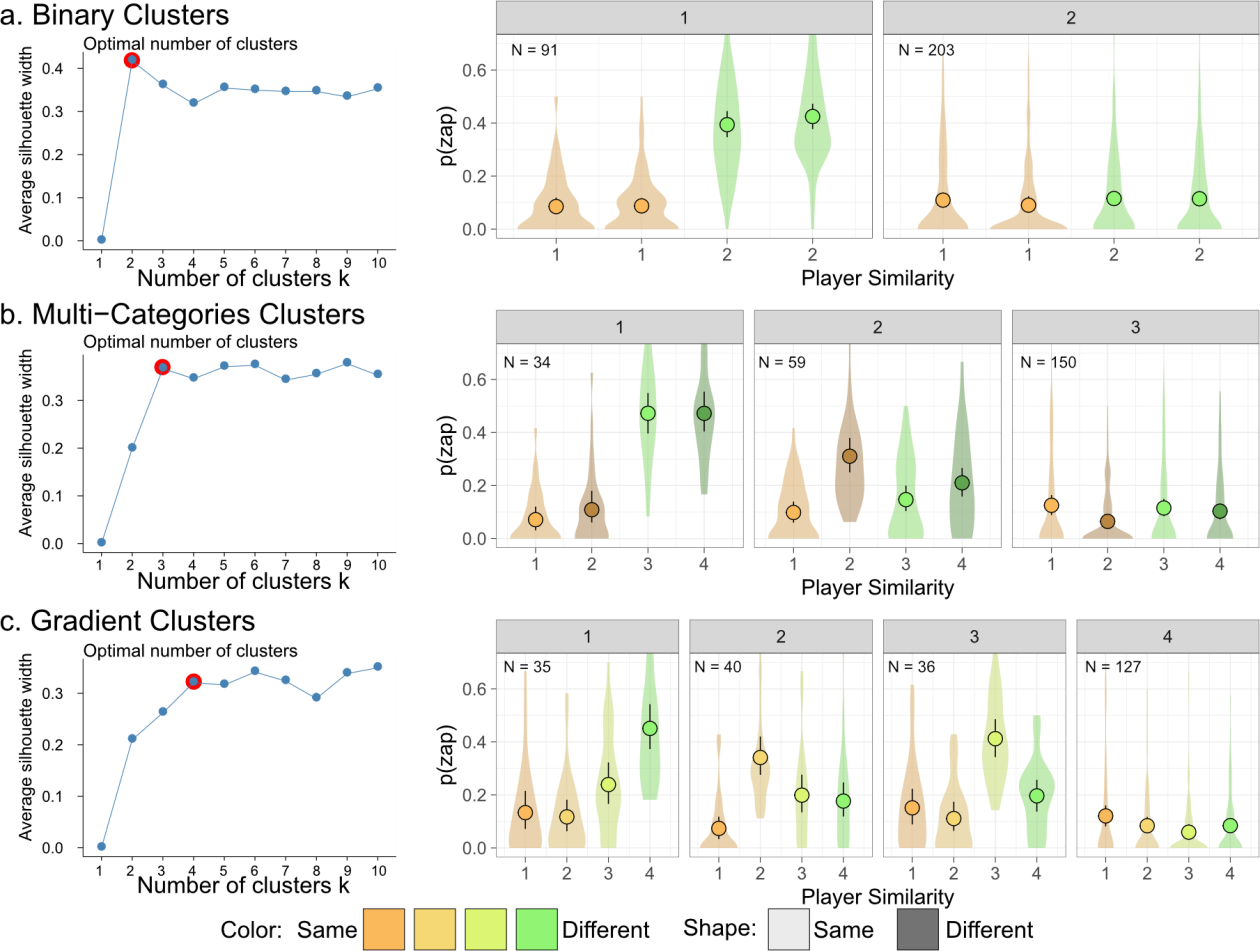
Cluster analysis revealed diverse rule-based categories in zapping behaviour. Left panels show silhouette analysis, with a red dot indicating the selected number of clusters. Right panels show the zapping behaviour of participants in each of the clusters, for each experimental condition. Circles represent the mean, lines represent bootstrap confidence interval of the mean, and violins represent the distribution of individual responses.

In a *k*-means clustering analysis, each participant’s likelihood to zap each of the other four players was used to examined how many clusters of zapping patterns could explain participants’ behaviour in each condition. Silhouette analysis was used to identify the contribution of each clustering number and evaluate the minimum number of clusters that captured the data [40]. As seen in figure 4, the explanatory benefit of increased number of clusters either peaked or plateaued in each of the experimental conditions, and the number of clusters was set according to this point of peak/plateau.

In the binary condition, the benefit of an increased number of clusters peaked at two clusters. About a third of our participants showed a clear colour effect (cluster 1, *n* = 91), while participants in the second cluster did not differentiate between players (cluster 2, *n* = 203; figure 4a). These results existed in both the all-zappers and all-avoiders experimental conditions (see electronic supplementary material, figure S1). This shows that participants’ social categorization relied solely on players’ colour, which was the only social marker available to them.

In the multi-categories condition, the benefit of an increased number of clusters plateaued at three clusters. One cluster of participants showed a clear colour effect (cluster 1, *n* = 34), the second cluster showed dependency on shape (cluster 2, *n* = 59) and the last cluster did not differentiate between players (cluster 3, *n* = 150; figure 4b; see results separated by all-zappers and all-avoiders in electronic supplementary material, figure S2). In the multi-categories condition, participants showed either sensitivity to colour or shape, based on the two social markers available to them.

Finally, in the gradient condition, the benefit of an increased number of clusters plateaued at four clusters. One cluster of participants showed increased zapping of the most different player (cluster 1, *n* = 35), another cluster singled out the second most similar player (cluster 2, *n* = 40) and yet another cluster singled out the third most similar player (cluster 3, *n* = 36). Another cluster of participants did not differentiate between players (cluster 4, *n* = 127; figure 4c; separated by all-zappers and all-avoiders in electronic supplementary material, figure S3). These results indicate that different subgroups of players followed different rule-based categorization, each showing differential treatment of a different player.

### (d) Intergroup bias in star allocation behaviour

Finally, participants performed the pre-registered star allocation task after playing the star-harvest game, a task similar to the dictator game used in many intergroup experimental designs [26,39,41]. Participants had ten extra stars that they could distribute between the four other players in the game. As all bot-players displayed the same behaviour during the game, star allocation was expected to be based only on social signals. To evaluate the use of social markers in each condition, a linear regression was used, with social markers as independent variables (same/different colour in the binary condition, same/different colour, same/different shape, and their interaction in the multi-categories condition, and similarity linear (0−3) and quadratic (0−9) distance in the gradient condition) and number of stars as the dependent variable.

**Figure 5.**
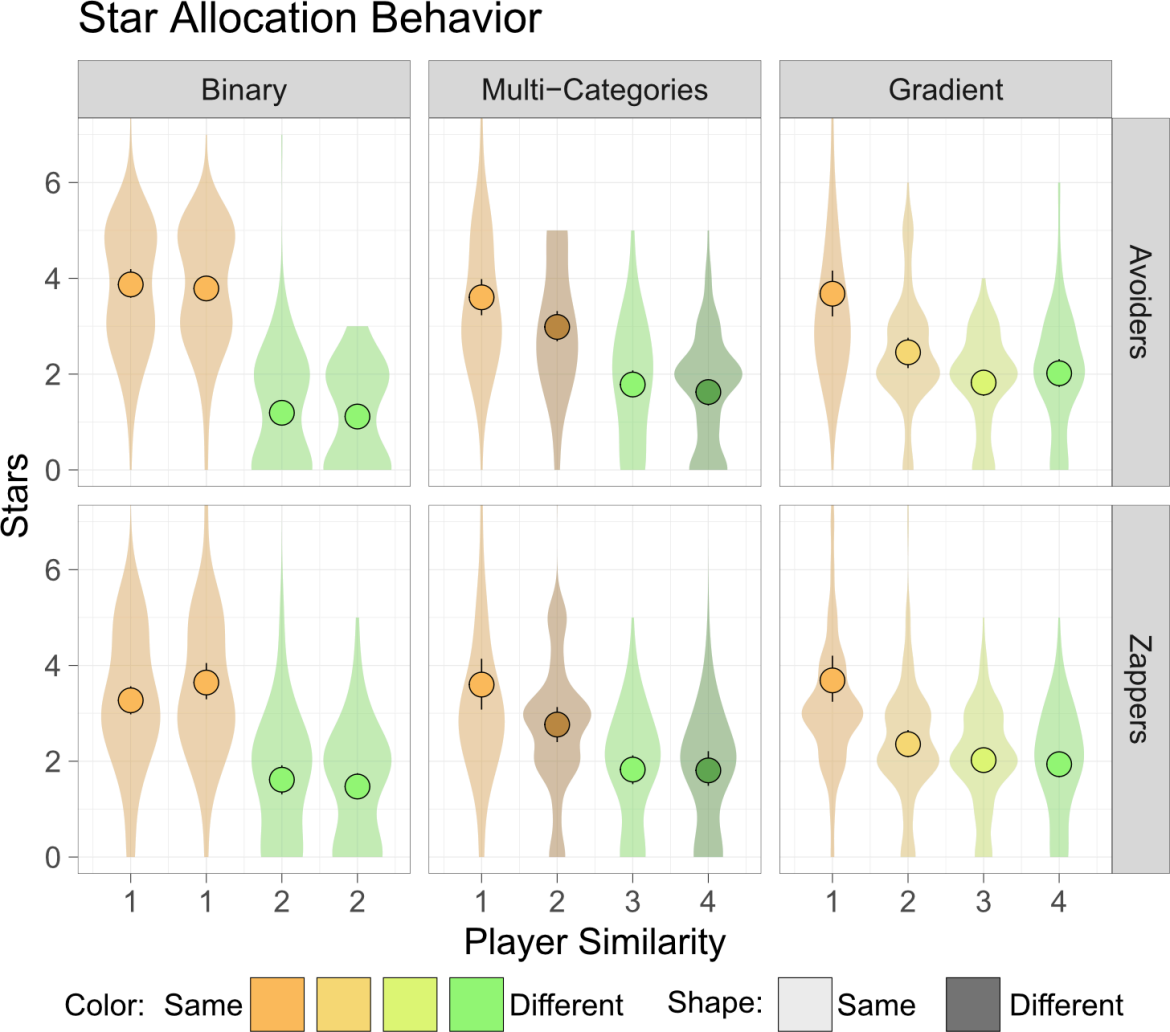
Post-task star allocations. Participants could allocate ten extra stars to the four other players after the experiment was over. Participants consistently gave more stars to players who resembled them than to dissimilar players. Participants used complex social markers when treating players who were relatively similar to them. Circles represent the mean, lines represent bootstrap 99% confidence interval of the mean and violins represent the distribution of individual responses.

In the binary condition, participants allocated significantly more money to players with the same colour as them than to those with different colour, both in the all-avoiders condition (2.67 ± 0.098, *t*(623) = 27.26, *p* < 0.001, SC: [95% CI] = 1.47 [1.37, 1.58]) and in the all-zappers condition (1.91 ± 0.12, *t*(558) = 15.21, *p* < 0.001, SC: 1.08 [0.94, 1.22]) (figure 5). In the multi-categories condition, participants were affected both by colour (all-avoiders: 1.36 ± 0.17, *t*(517) = 8.06, *p* < 0.001, SC: 0.85 [0.65, 1.06], all-zappers: 0.95 ± 0.22, *t*(456) = 4.34, *p* < 0.001, SC: 0.52 [ 0.29, 0.76]), and by the interaction between shape and colour (all-avoiders: 0.46 ± 0.24, t(517) = 1.93, *p* = 0.053, SC: 0.29 [0.00, 0.59], all-zappers: 0.82 ± 0.31, t(456) = 2.62, *p* = 0.009, SC: 0.45 [0.11, 0.78]). These effects indicate that participants gave more stars to same-colour players and extra stars to players who had the same colour and shape as them (marginally so in the all-avoiders group). Finally, in the gradient condition, there was a significant negative effect of linear colour similarity (all-avoiders: −2.33 ± 0.35, *t*(446) = −6.7, *p* < 0.001, SC: −1.61 [−2.09, −1.14], all-zappers: −2.11 ± 0.33, *t*(512) = −6.39, *p* < 0.001, SC: −1.45 [−1.89, −1.00]) and a significant positive quadratic colour-similarity effect (all-avoiders: 0.35 ± 0.07, *t*(446) = 5.17, *p* < 0.001, SC: 1.24 [ 0.77, 1.72], all-zappers: 0.31 ± 0.065, *t*(512) = 4.78, *p* < 0.001, SC: 1.08 [0.64, 1.53]). The combined effect of linear and quadratic similarity indicates that participants gave more stars to players who resembled them, but this tendency diminished with increased dissimilarity, leading to conservative-rule pattern of star allocation. These results suggest that participants used complex social markers when treating players who were relatively similar to them (same or similar colour) but stopped using them at some dissimilarity point, regarding all other players similarly.

## 4. Discussion

In a set of three experiments, a re-analysis of a previous experiment and two new experiments using a star-harvest multi-player game, the complexity of social markers was manipulated to examine how it affected social categorization. Participants showed intergroup bias in their zapping behaviour in all three experimental conditions, zapping players who resembled them less than dissimilar players. However, this effect was smaller when social markers were complex, either multidimensional or continuous. In addition, participants showed reduced intergroup bias during the experimental block in the gradient condition, where players’ colours lie on a continuum. When examining whether participants used binary rules in their zapping behaviour, group-level results indicated that on average participants were sensitive to multiple social markers in their behaviour. However, clustering analysis showed that this group average was the result of a mixture of different binary-rule clusters. Participants in the multi-categories condition showed either a shape- or colour-based binary decision pattern, and participants in the gradient condition showed multiple binary rules that singled out different players. These results indicate that participants tended to follow a less cognitively demanding binary rule in their categorization, and that increased complexity of social markers allowed the appearance of multiple individual rules, which contributed to overall reduction of intergroup bias.

Participants tended to adopt idiosyncratic binary rules, rather than more subtle continuous zapping patterns, that were dependent on the affordances of the experimental task. When shape dimension was presented in addition to the colour dimensions, some participants followed a colour-rule and some a shape-rule, zapping players whose shape differed than theirs more than similarly shaped players, but there was no cluster of intersection zapping pattern. In the gradient condition, participants tended to zap one player more than others, either one of the intermediate players or the most different player. These findings are in line with previous works showing a tendency to form binary categorizations in complex settings in non-social categorization [28,34] and social categorization [14,29]. This may reflect calculated use of cognitive resources [42,43]. When playing the game, participants must process and respond to multiple signals, such as the location of stars and other players, and evaluating players’ similarity to determine zapping likelihood is cognitively demanding, leading participants to rely on more simple binary rules. However, when the game is over and participants were faced with a single star-allocation task, they were more nuanced in their behaviour and showed intersection consideration, in line with previous findings in multi-dimension groups [25,26]. These findings therefore support the notion that cognitive resource management may favour the use of simple heuristics in social categorization, especially during demanding tasks.

While participants tended to use binary categories, they varied in their specific categorization rules. Rule diversity was highest in the gradient condition, which allowed participants to single out different players for differential treatment. These diverse rules led to overall reduction in average, group-level, intergroup bias. In addition, when participants played with players with high diversity on the colour dimension, they showed a reduction in their differential treatment of players throughout the experiment. These findings indicate that complex social settings, where markers of social identity are complex and multidimensional, allow individuals to parse or divide them into different categories in many ways, along different dimensions. This can make unified categories less salient on the population level, as each individual adopts a different category-based behavioural pattern. Social dimensions with high variability may also be less salient on the individual level, making them less relevant for social categorization. These results are in line with findings showing that societies with diverse populations display lower intergroup differentiation, seeing different groups as more similar to each other, and that stereotypes were found to be most distinct for individuals with the lowest exposure to ethnic diversity [44]. In another work, the presence of individuals with dual identity, belonging to two groups, can serve as a gateway to reducing intergroup tensions [45]. The current findings provide a categorization perspective for these works—when social markers are more complex, they lead to reduced saliency of social groups on the group and individual level.

The observed diversity of rules and reduction in intergroup bias align with the literature on identity signalling and its relation to ingroup cohesion and hostility towards outgroups. Computational simulations and experimental works show that cooperation deteriorates when social categories are uncertain [46–48]. When people need to cooperate but fear defection, signalling one’s similarity to the other party can facilitate cooperation [49]. These can contribute to the formation of norms regarding identity signalling and make people more likely to cooperate with ingroups and less likely to cooperate with out-groups [47,50]. People seem to use identity signalling in strategic and sophisticated ways, making sure that their signals are received by the right target [51]. This pattern can actively make social categories salient and binary, removing complex and intermediate social markers. By removing complexity, social categories become less idiosyncratic, more salient across populations and more relevant for guiding individuals behaviour. This can also explain hostility to intermediate groups, which poses a challenge to categorization as a solution for cooperation problems [30,48].

The current findings describe cognitive mechanisms and environmental settings that can alleviate intergroup bias. While contact theory of intergroup bias suggests that social interactions with outgroups can reduce intergroup conflict, it is mostly limited to cooperative settings [35,36]. In addition, previous work showed that intergroup bias may persist even after direct interaction, due to confirmation bias [4,37]. The current findings suggest that increased complexity of social markers allows people to overcome their confirmation bias and judge others more by their actions and less by their group identity, even in competitive settings. This can have clear implications for the study of real-world intergroup settings, such as intergroup conflicts and polarization, and the design of conflict resolution programs. Intergroup conflicts and tensions are often not binary conflicts between two distinct groups, but are more complex, including intermediate subgroups and continuous and intersecting differences in values [12,52]. However, when studying conflict and polarization, surveys and experiments often focus on binary categories (e.g. conservatives/liberals). Providing participants with more complex social markers—for example, where people stand on an array of topics and values—may provide a richer description of polarization processes and intergroup conflicts. Building on the cognitive insights shown here, future work could examine ways to relieve social conflict and polarization by embracing complexity. Highlighting the complexity of social categories, which is more cognitively demanding, may reduce their saliency and importance in individual decision-making.

The current work used a novel and engaging experimental design and measures, and was inspired by findings from cognitive psychology. This required some design choices that could limit the interpretation of the results. Most notably, participants did not play with other participants but with algorithmic-players. While algorithmic-players’ identity was revealed only after the task, participants may have shown different patterns of behaviour if they had been sure that they were playing with other human participants (but this is not certain—see previous discussion [4]). The use of algorithmic-players allowed control of the players’ behaviour regardless of their appearance, which was important for the interpretation of participants’ zapping behaviour as dependent on social categories and not on players’ behaviour. However, this meant that it was not possible to detect dynamic changes in collective behaviour that are related to the saliency of social categories, for example whether participants converge over time to a unified social category or whether other signals of identity, such as proximity, emerge over time. These important questions should be examined in live interacting groups in future studies. In addition, using video game settings was engaging and allowed social behaviour to emerge in complex environmental settings, but could also bias participants. As many video games include conflicts and battles between teams, participants could assume that this was a similar case. However, this consideration should have affected all experimental conditions, and therefore differences in intergroup relations are still meaningful. It is worthwhile to explore other types of games, such as cooperative games, in future studies.

To conclude, this work asked how complex social markers affect social categorization. Building on findings from social and cognitive psychology, which examined how multiple dimensions and continuous markers shape the content of social categories, this work manipulated social markers and examined how it affected intergroup bias. The main findings show that in environments with complex social markers, intergroup bias is attenuated across participants and over time within participants. This effect relies on participants displaying idiosyncratic rule-based binary social categorizations. When social markers were variable or multidimensional, participants could use them to categorize players in different ways, making overall social categories less salient. These findings highlight the importance of cognitive resource limitations for understanding the emergence of collective behaviour and provide an explanation to the way variability in social markers can lead to diversity of social relationships and reducing stereotype and intergroup bias.

## Data Availability

Pre-registration and data availability pre-registrations can be accessed for the multi-categories condition [53] and the gradient condition [54]. A demonstration of the experimental task is available at https://socialdecisionlab.net/stuff/GridWorldDemoN/. Data and scripts are available at OSF [55]. Supplementary material is available online [56].
